# Inter-observer variability of LV mass and volumes with compressed sensing

**DOI:** 10.1186/1532-429X-16-S1-W37

**Published:** 2014-01-16

**Authors:** Suzanne Lydiard, Andreas Greiser, Michaela Schmidt, Mariappan S Nadar, Brett R Cowan, Alistair Young

**Affiliations:** 1 Auckland MRI Research Group, University of Auckland, Auckland, New Zealand; 2 Healthcare Sector, Siemens AG, Erlangen, Germany; 3 Siemens Corporate Technology, Princeton, New Jersey, USA

## Background

Recently developed compressed sensing (CS) SSFP sequences significantly reduce data acquisition time thereby reducing patient breath-hold and scan time relative to standard techniques. The variability of ventricular functional (VF) analysis of CS data is not known so this study investigated the inter-observer variation of left ventricular (LV) mass and volume measurements compared to standard SSFP acquisitions.

## Methods

Twenty healthy human subjects (9 male, 40 ± 14 years) underwent LV SSFP imaging on a MAGNETOM Skyra 3T scanner (Siemens, Germany). Three sequences were acquired (i) gold standard fully sampled SSFP (FULL) and two 2D prototype sequences featuring CS reconstruction and regularisation in space and time with acceleration factors (ii) R = 4 (R4) or (iii) R = 9.2 (R9.2). 5-8 short axis slices (thickness 6 mm, slice gap 9 mm) and three long axis slices (4-,3-,2-chamber), FOV= 260-340 mm, were acquired for each sequence. FULL images were acquired over 14 heart-beats with TE = 1.54 ms, α = 51°, 25 frames, matrix 256 × 256 and iPAT factor 2. R4 images were acquired over 4 beats with TE = 1.29 ms, α = 41°, 21 frames, matrix 192 × 143 with iPAT. R9.2 images were acquired over 2 beats (one dummy beat for steady state preparation, thereby representing 'realtime' acquisition) with TE = 1.27 ms, α = 42°, 19frames, matrix 192 × 129. Images were reconstructed on-line using a non-linear iterative CS method with k-t regularisation derived from a SENSE type reconstruction [1]. LV function parameters were calculated by two analysts using CIM Version 7, blinded by sequence type and participant. The percentage differences between the two analysts' results were calculated to give an indication of inter-observer variability.

## Results

The average of both analysts' measurements over all participants for the FULL acquisition were: end-diastolic volume (EDV) = 14.6 mL, end-systolic volume (ESV) = 56.9 mL, ejection fraction (EF) = 61.4 mL and LV mass (LVM) = 113.9 g. The mean difference (bias) between the analysts is provided in table 1 and show a similar trend for all acquisitions likely related to the preference of each analyst for the positioning the endocardial and epicardial contours. While there were significant differences (p < 0.05) in all but EF for the CS sequences, these were of a comparable magnitude across all three sequence types. This suggests the bias is related primarily to the analyst rather than the accelerated CS acquisitions.

**Table 1:** Inter-observer percentage differences (± standard deviation) for ventricular function measurements for CS acceleration.

	EDV	ESV	EF	Mass
	**Inter-observer % Diff**	**Inter-observer % Diff**	**Inter-observer % Diff**	**Inter-observer % Diff**

FULL	2.1 ± 2.8*	2.5 ± 6.0**	-0.4 ± 3.9*	-8.9 ± 3.5**

R4	1.3 ± 2.6*	3.3 ± 5.6*	-1.5 ± 3.6	-8.9 ± 5.8**

R9.2	1.7 ± 3.3*	6.0 ± 5.5*	-3.8 ± 4.2	-8.7 ± 5.7**

p values represent repeated measures Bonferoni corrected ANOVA, * represents 0.05 < p < 0.01, ** represents p < 0.01

## Conclusions

The inter-observer variability of LV function for CS data was comparable to that for standard acquisitions. Inter-observer variability of VF was not generally increased when CS was applied to SSFP cine analysis

## Funding

Siemens Medical Systems.

**Figure 1:**
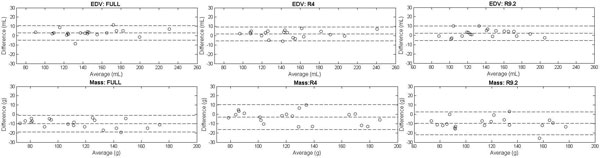
**Bland-Altman analysis of EDV (top row) and Mass (bottom row) comparing the two analysts for FULL (left), R4 (middle) and R9.2 (right) sequences**.
